# Implications of Von Hippel-Lindau Syndrome and Renal Cell Carcinoma

**DOI:** 10.15586/jkcvhl.2015.41

**Published:** 2015-09-25

**Authors:** Kenan Ashouri, Sophia Mohseni, John Tourtelot, Pranav Sharma, Philippe E. Spiess

**Affiliations:** 1 Department of Genitourinary Oncology; 2 Department of Endocrinology, Moffitt Cancer Center, Tampa, FL, USA.

## Abstract

Von Hippel-Lindau syndrome (VHLS) is a rare hereditary neoplastic disorder caused by mutations in the vhl gene leading to the development of tumors in several organs including the central nervous system, pancreas, kidneys, and reproductive organs. Manifestations of VHLS can present at different ages based on the affected organ and subclass of disease. In the subclasses of VHLS that cause renal disease, renal involvement typically begins closer to the end of the second decade of life and can present in different ways ranging from simple cystic lesions to solid tumors. Mutations in vhl are most often associated with clear cell renal carcinoma, the most common type of renal cancer, and also play a major role in sporadic cases of clear cell renal carcinoma. The recurrent, multifocal nature of this disease presents difficult challenges in the long-term management of patients with VHLS. Optimization of renal function warrants the use of several different approaches common to the management of renal carcinoma such as nephron sparing surgery, enucleation, ablation, and targeted therapies. In VHLS, renal lesions of 3 cm or bigger are considered to have metastatic potential and even small lesions often harbor malignancy. Many of the aspects of management revolve around optimizing both oncologic outcome and long-term renal function. As new surgical strategies and targeted therapies develop, the management of this complex disease evolves. This review will discuss the key aspects of the current management of VHLS.

## Introduction

Von Hippel-Lindau syndrome (VHLS) is a rare, but highly penetrant, autosomal dominant hereditary neoplastic disorder caused by a genetic mutation in the vhl gene (vhl) that occurs in roughly 1 in 36,000 births (1). Mutations in vhl can be deletions or missense in the short arm of chromosome 3, resulting in an increased propensity to develop a variety of benign and malignant tumors, such as hemangioblastomas in the central nervous system (CNS), renal cysts and renal cell carcinomas (RCC), pheochromocytomas, pancreatic islet cell tumors, endolymphatic sac tumors, and both broad ligament and epididymal cystadenomas in women and men, respectively (1, 2). The disease is classified into type 1, the result of a nonsense mutation or deletion, and type 2, the result of a missense mutation, based on adrenal involvement and risk of pheochromocytoma, in which type 2 carries risk of pheochromocytoma and type 1 does not (3, 4). Type 1 typically carries high risk for renal involvement, high risk for CNS hemangioblastomas, and low risk for the development of pheochromocytomas (1). Type 2 VHLS is further divided into A, B, and C based on the predisposition for renal involvement, classified as low risk, high risk, and no risk, respectively (3, 4). Type 2A typically carries low risk for renal involvement and high risk for the development of both hemangioblastomas of the CNS and pheochromocytomas. Type 2B typically carries high risk for renal involvement, CNS hemangioblastomas, and pheochromocytomas. Type 2C typically carries very low risk for renal involvement, low risk for CNS hemangioblastomas and high risk for pheochromocytomas (1). This review will discuss the implications of renal involvement in VHLS and therefore only include VHLS type 1, type 2A, and type 2B.

## Materials and Methods

A comprehensive literature review was conducted for articles published between 1990 and 2015 using PubMed with regard to genetic and molecular mechanisms of vhl and the clinical and pathological diagnosis, treatments, and prognosis of renal tumors in this disease. Data for this review was acquired using combinations of the following terms: von Hippel-Lindau syndrome, von Hippel-Lindau disease, von Hippel-Lindau, renal cell carcinoma, radiology, screening, treatment, nephrectomy, VHL, VHL gene, HIF, metastatic, nephron sparing surgery, ablation. We excluded articles not written in English and any study that excluded hereditary cancer syndromes from its inclusion criteria, which resulted in the exclusion of approximately 10 peer-reviewed articles each. After assessing the available literature, 81 peer-reviewed articles and their sources met our inclusion criteria and were included in this review.

## Genetics of vhl and Molecular Mechanisms of VHL protein

The vhl gene is located at chromosome 3p25-p26, and contains 3 exons; over 300 germ-line mutations have been described leading to VHLS (1, 2). The vhl gene is also conserved across many taxa of living organisms and thus is considered to be fundamental to life (5). Several studies have implicated the role of vhl as a tumor suppressor gene and mutations of the wild-type and loss of heterozygosity are thought to play a major role in tumorigenesis (6).

The protein product of vhl, (pVHL), plays a major role in the ubiquitination of multiple proteins involved in many cellular pathways and contains binding domains for elongin C, elongin B, and cullin-2 (7–9). The resulting multimeric protein complex is known as the VHLcomplex (7). Often mutations in vhl affect the expression of pVHL, but some missense mutations can affect pVHL and elongin C binding (10). The role of hypoxia induced factor (HIF), a helix-loop-helix transcription factor that plays a major role in the cellular response mechanism to hypoxia, in the VHL pathway has been well-documented (11–14). The functional, dimeric form of HIF is comprised of 2 subunits, an HIFα subunit (either HIF1α, HIF2α, or HIF3α) and an HIF1β subunit (15, 16). HIFβ is constitutively expressed in contrast to HIFα, whose expression is tightly regulated via ubiquitination by the VHL complex and proteasomal degradation (9). Under normoxic conditions the VHL complex recognizes HIFα, which is polyubiquinated and degraded, thus reducing cytoplasmic HIFα (17). This recognition of HIFα for ubiquitination by VHL complex requires hydroxylation of proline residues on HIFα subunits by prolyl hydroxylase enzymes (PHD1, PHD2, PHD3), which require oxygen as a cofactor (7, 8, 18, 19). Thus, hydroxylation of HIFα proline residues and subsequent ubiquitination occurs only in the presence of oxygen (20). Under hypoxic conditions, hydroxylation of HIFα prolines does not proceed, preventing or reducing the VHL complex recognition of HIF for ubiquitination, leading to the accumulation of HIFα, making it available to bind constitutively expressed HIFβ (21, 22). This new HIFαβ complex passes into the nucleus and binds to sequences known as hypoxia response elements that regulate of over 800 hypoxia inducible genes (23). Under normal conditions, expression of these hypoxia inducible genes allows a normal cellular adaptive response to hypoxia; however the aberrant regulation of many of these genes plays a major role in tumorigenesis and angiogenesis. The role in tumorigenesis of these genes varies. Several hypoxia inducible genes such as vascular endothelial growth factor (VEGF), erythropoietin, and platelet-derived growth factor (PDGF) have been found to have roles in angiogenesis (21, 24). Other targets of the pVHL-HIF pathway include glucose uptake via glucose transporter 1 (GLUT1), extracellular matrix remodeling via E-cadherin and matrix metalloproteinases (MMPs), microtubule stabilization via modulation of the primary cilium, regulation of apoptosis via p53, cell proliferation via transforming growth factor-α (TGFα) and epidermal growth factor (EGFR), and cell cycle progression via cyclin D1 (25–31). More recent studies have elucidated the role of mutations in other genes coding for proteins involved in the VHL-HIF pathway, such as TECB1 coding elongin C, resulting in similar aberrancies in the pVHL-mediated regulation of HIF and downstream induction of hypoxia inducible genes (32).

Several studies have also elucidated other roles of pVHL in HIF-independent pathways involved in tumorigenesis including the modulation of extracellular matrix components such as fibronectin, collagen IV, and integrins (33–35) Furthermore, pVHL has been found to be involved in the non-HIF-mediated stabilization of microtubules through a pVHL binding protein (VBP-1) by impacting the assembly of microtubule subunits (11, 18, 36).

## Renal Pathology and VHLS

VHLS is the most common cause of hereditary renal cell carcinoma (RCC), specifically clear cell renal carcinoma (ccRCC) (37). Prior to the use of computed tomography (CT) in regular surveillance of VHLS, metastatic ccRCC was thought to be the leading cause of death in VHL patients (38). VHLS-associated RCC comprises about 4% of the total RCC incidence and is equal in both males and females (5, 39). While renal cysts are estimated to occur in up to 63% of patients with VHLS, renal tumors are estimated to occur in 25–45% of patients with VHLS and have a mean age at presentation of 39 years (5, 37). It should be noted that even cases of sporadic, non-familial ccRCC involve mutations or methylation of vhl in up to 91% of cases (40). Renal tumors less than 3 cm tend to be less invasive with lower pathological T stage; however these tumors tend to become more aggressive as they increase in size (41). Growth rates of renal tumors vary from 0.2 cm/year to 2.2 cm/year with a mean of 1.6 cm/year and a mean volume doubling time of approximately 26 months (42, 43). Metastasis is rare in tumors under 3 cm and tumor size on CT has been shown to be a prognostic indicator of overall survival (5, 41).

Renal involvement in VHLS is typically characterized by multiple, bilateral, recurring cysts or tumors (37, 38). Previous studies identified an average of approximately 7 renal cysts and 3 renal tumors per kidney, however it is hypothesized that the number of microscopic foci of malignant and non-malignant neoplasms could be in the hundreds and thousands, respectively (44, 45).

Renal cysts can be classified as simple or complex, depending on the presence and pattern of septation. The Bosniak classification scale is useful in describing radiologic findings in VHLS, but due to the high potential for malignancy of VHL cysts, its prognostic use in management is limited (39). Simple sporadic renal cysts have a very low rate of malignancy under the classic Bosniak classification system, whereas in VHLS simple cysts often already contain malignant characteristics in their walls (45). For this reason, it is recommended to undergo screening and active surveillance every 6 to 12 months depending on the rate of tumor growth and the patient specific history of malignancy (46).

## Radiology of renal masses in VHL

Renal masses in VHLS often present as cystic masses on CT with varying degrees of complexity and septa. Classically, the Bosniak classification scale provides a method of assessing the malignant potential of a renal cyst based on the radiologic characteristics of the cyst and provides diagnostic indications for management (47). It was initially described in the context of CT, but can also be used for MRI and has also been applied to ultrasound (47, 48). As stated above, it is important to note that recommendations for management of renal cysts cannot be made based on the Bosniak classification due to the fact that VHL renal cysts tend to have foci of cellular malignancy in their walls (46). For this reason, even simple cysts are likely to contain some malignancy and require active surveillance.

The Bosniak system is useful in stratifying the variation of presentations of renal lesions in VHLS, but management decisions should be based on the consistency of the lesion and size of the largest mass (41). Even larger masses, such as a category III, may necessitate active surveillance instead of treatment based on the consistency of the lesion and number of surgeries in the patient’s history (39). The use of ultrasound has proven useful in the surveillance of lesions and identifying their consistency, however, this technique lacks uniformity and is user dependent. CT and MRI are the most useful imaging modalities for the surveillance of renal lesions of VHLS (39).

The feasibility and safety of regular CT scan in patients with VHLS has come into question considering that MRI is a better modality for monitoring and identifying several non-renal tumors common to VHLS and that high levels of radiation exposure can occur with regular CT (46). Several studies suggest the use of regular MRI in surveillance of VHL lesions; however these advantages are not without risk (49, 50). The possibility of nephrogenic systemic fibrosis secondary to the use of gadolinium contrast in patients with compromised renal function and cost should be considered in surveillance strategy (50). For this reason, it is important to consider each patient on a case-by-case basis, but active surveillance has been well established as a key aspect of the management of VHLS (39).

## Treatment of renal masses in VHLS

Over the past few decades, nephron-sparing surgery (NSS), either open partial nephrectomy (PN) or enucleation, has become the standard surgical intervention for managing RCC in VHLS (51–53). Prior to NSS, the major approach was bilateral radical nephrectomy, leaving patients dependent on transplantation or dialysis, with high mortality rate and low quality of life (54). In 1995, Steinbach et al (54) found cancer-specific survival (CSS) rates at 5 and 10 years of 100 and 81 percent for patients undergoing NSS, compared to the CSS rates at 5 and 10 years of 73 and 36 percent for patients undergoing radical nephrectomy. Since then other studies have identified NSS as a superior approach for most cases (55, 56). The goal of NSS is to maximize remaining functional renal parenchyma (56). Since smaller renal tumors are typically considered low stage and low grade with low metastatic potential, PN is usually reserved for tumors greater than 3 cm, at which metastatic potential drastically increases, however some centers prefer a 4 cm cutoff (42, 57). The de novo recurrence rate of RCC in VHL is up to 85% at 10 years and the need for multiple NSSs will arise in many patients (54). It should be noted that in 2007, Ploussard et al (58) found a cancer-specific survival of 93.8% in a 24 VHL patient cohort in which 63.4% of patients required repeat NSS. A more recent study in 2012 by Singer et al (55) found a cancer specific survival at 10 years of 97% in 128 patients who underwent at least bilateral NSS, of which 70% had VHL and 68% of patients required repeat ipsilateral NSS. In 2012, Jilg et al (57) also found repeat NSS to be practical, establishing a limit of up to three repeated NSSs with complication rates of 53.6% for the first NSS, 33.3% for the second NSS, and 67% for the third NSS with a high proportion of more severe complications after the third NSS. Furthermore, NSS has been proven to be superior to bilateral radical nephrectomy and renal replacement therapy in overall survival, cancer-specific survival and morbidity and mortality in the management of VHLS (55). However, this study and other studies highlight the technical skill requirement and difficulty of repeat NSS (55, 59, 60). The number of repeated surgeries is limited by post-operative fibrosis and the increased morbidity and mortality associated with each surgery (53, 56, 61).

The recurrent nature of this disease obviates the need for a less invasive second-line therapy (60). Percutaneous ablation, including radiofrequency ablation and cryoablation, has proven to be a major candidate in both early and later stages of management of smaller renal tumors in VHLS (61–65). Percutaneous ablation is typically reserved for tumors between 2–3 cm and as a secondary procedure in most cases (42, 65). In 2010, Park et al. (66) found that radiofrequency ablation successfully managed 88% of renal masses in 11 patients with recurring tumors secondary to VHLS. In 2013, Yang et al (67) found cryoablation to have a failure rate of 7.7% in patients with VHLS compared to a failure rate of 21.4% for radiofrequency ablation. They also found both radiofrequency ablation and cryoablation to have 100% 5-year CSS (67). Although recurrence rates are higher than NSS, ablation carries lower incidence of morbidity and mortality (66). However, there are several contraindications to ablation including cystic tumors, tumors adjacent to critical structures (e.g. ureter and bowel) and extensively multifocal tumors, all of which are common in VHLS (60).

Several surgical interventions are available for the management of RCC in VHLS and weighing oncologic outcomes against operative morbidity and mortality is crucial in VHL management decisions (60). However, trends in preserving functional renal parenchyma in patients with VHL have improved survival outcomes over the past few decades (56). Pre-operative management and surveillance of other malignancies must also be considered, as these can cause major intraoperative complications, such as hemorrhage of a hemangioblastoma or pheochromocytoma disrupting patient intraoperative stability (53). It is important to keep in mind that these patients are not only subject to RCC but also multifaceted systemic malignant neoplasms, the treatment of which can have high morbidity and severely decrease quality of life (53). Maximizing efforts to use minimally invasive techniques should be a major consideration of future management.

## Targeted therapy in RCC and VHL

Advancements in understanding vhl and the activity of pVHL have provided key insight into potential mechanisms of therapeutic intervention in ccRCC and systemic treatment for VHL using targeted therapies (68). Few studies have been conducted on the efficacy of these drugs specifically in the context of VHSL, however, extensive research has been conducted on metastatic ccRCC. Considering most ccRCC involves aberrancies in vhl, these studies are expected to translate (40).

Older targeted therapies involved immunomodulation through cytokines IFN-á and IL-2, and had low relative risk reduction. IFN-á proved to have little therapeutic value and caused flu-like symptoms, fatigue, and depression; however, it is still seen in some trials as a point of reference (69). IL-2 showed benefit in some patients for long-term use and is still recommended in few select cases, however the adverse events of IL-2 have limited its use in current practice (70). These more classic immunotherapies have given way to newer targeted therapies involving the VEGF and mammalian target of rapamycin (mTOR) pathways that have since been approved by the Food and Drug Administration (FDA).

As noted previously, the loss of functional pVHL results in downstream over-expression of VEGF (21). Several oral tyrosine kinase inhibitors (TKI), sunitinib, sorafenib, pazopanib, and axitinib, and one monoclonal antibody, bevacizumab, decrease VEGF and PDGF signaling thereby decreasing angiogenesis and disrupting tumorigenesis (71). In 2009, Motzer et al (72) found a significantly improved overall survival and progression free survival (PFS) using Sunitinib as a first-line treatment in patients with metastatic ccRCC. Another study by Jonasch et al (73) found a 33% partial regression of RCC in a cohort of 18 patients with VHLS (p=0.014). Sorafenib, a TKI similar to sunitinib, as first-line treatment has also been proven to improve PFS in metastatic ccRCC, however, both sunitinib and sorafenib have similar adverse event profiles including diarrhea, rash, fatigue, and hand-foot syndrome (72, 74). Early trials with pazopanib found a significantly increased PFS with a better adverse event profile than other TKI anti-angiogenics, however more recent trials have shown no significant increase in overall survival over placebo (75, 76). Bevacizumab, a humanized monoclonal antibody for VEGF-á, has shown increased PFS in second-line use with IFN-á (77). Many other TKIs are under investigation for use in ccRCC, but have not yet been approved by the FDA (71).

Furthermore, the FDA has approved two agents, temsirolimus and everolimus, for ccRCC that involve the disruption of the PI3K/AKT/mTOR pathway. Temsirolimus, an inhibitor of mTORC1, is an intravenous agent that has shown significantly improved survival and can be used among high-risk patients (78, 79). Everolimus, an oral mTOR directed therapy, has been approved for second-line use in metastatic ccRCC, showing improved PFS, but no overall survival improvement (80, 81). Both of these agents have a similar adverse event profile of rash, mucositis, interstitial pneumonitis, hyperglycemia, and hyperlipidemia (78, 80).

## Conclusion

In conclusion, VHLS is a multifocal, hereditary neoplastic disorder that necessitates a complex multidisciplinary approach in its management (53). The protein product of vhl, known as pVHL, forms a multimeric complex with other intracellular proteins to form a molecule capable of ubiquitinating and degrading HIF proteins, preventing the over-expression of hypoxia inducible genes. Aberrant VHL contributes to angiogenesis and tumorigenesis and is implicated in both sporadic and VHLS-associated ccRCC (7–32). Tumors can be cystic or solid in nature and a cutoff of typically 3 cm, but sometimes 4 cm, is used to indicate the necessity for surgical management (42, 57). Radiologic surveillance can be done via several modalities including ultrasound, MRI, and most often CT, however each modality has its own advantages and disadvantages (39). NSS is the preferred surgical approach for renal tumors in VHL, typically using open PN for earlier, larger masses and percutaneous ablation for later, smaller tumors (55, 66). Systemic therapy, involving TKIs of the VEGF pathway and mTOR inhibitors, has been shown to improve CSS in metastatic ccRCC and in the rare studies available on VHL only cohorts (71–81). The multifocal nature of VHL mandates complex multidisciplinary management strategies that require special considerations (53).
